# Independent iron and light limitation in a low-light-adapted *Prochlorococcus* from the deep chlorophyll maximum

**DOI:** 10.1038/s41396-020-00776-y

**Published:** 2020-09-23

**Authors:** Nicholas J. Hawco, Feixue Fu, Nina Yang, David A. Hutchins, Seth G. John

**Affiliations:** 1 Department of Earth Sciences, University of Southern California, Los Angeles, CA USA; 2 Marine and Environmental Biology, University of Southern California, Los Angeles, CA USA; 3 Present Address: Department of Oceanography, School of Ocean and Earth Sciences and Technology, University of Hawaiʻi at Mānoa, Honolulu, HI USA

**Keywords:** Biogeochemistry, Microbial ecology

## Abstract

Throughout the open ocean, a minimum in dissolved iron concentration (dFe) overlaps with the deep chlorophyll maximum (DCM), which marks the lower limit of the euphotic zone. Maximizing light capture in these dim waters is expected to require upregulation of Fe-bearing photosystems, further depleting dFe and possibly leading to co-limitation by both iron and light. However, this effect has not been quantified for important phytoplankton groups like *Prochlorococcus*, which contributes most of the productivity in the oligotrophic DCM. Here, we present culture experiments with *Prochlorococcus* strain MIT1214, a member of the Low Light 1 ecotype isolated from the DCM in the North Pacific subtropical gyre. Under a matrix of iron and irradiance matching those found at the DCM, the ratio of Fe to carbon in *Prochlorococcus* MIT1214 cells ranged from 10–40 × 10^−6^ mol Fe:mol C and increased with light intensity and growth rate. These results challenge theoretical models predicting highest Fe:C at lowest light intensity, and are best explained by a large photosynthetic Fe demand that is not downregulated at higher light. To sustain primary production in the DCM with the rigid Fe requirements of low-light-adapted *Prochlorococcus*, dFe must be recycled rapidly and at high efficiency.

Since its emergence several hundred million years ago, the *Prochlorococcus* genus has diversified into dozens of distinct subpopulations, but the main branches of this radiation (ecotypes) are structured primarily by their growth at different irradiance [1]. Upregulation of the photosynthetic apparatus in low-light-adapted ecotypes compensates for dwindling light flux, contributing to the formation of a DCM layer at the base of the euphotic zone [2]. As photosynthetic proteins represent a large pool of Fe in these cells, growth at low light is expected to increase photosynthetic Fe requirements [3], an effect that has been documented in temperate diatoms and other model phytoplankton [4, 5]. The stratification of *Prochlorococcus* ecotypes therefore implies a stratification of Fe requirements, such that low light at depth would be coupled to very high Fe demand.

Large Fe requirements may lead to Fe stress in the DCM, where dFe can fall below 10^−10^ mol L^−1^ (100 pM [6]). Incubations in the California Current have shown that diatoms and other eukaryotic phytoplankton at the DCM respond to increases in both Fe and light [7], but these taxa are less abundant in offshore waters. At Station ALOHA—a site that is broadly representative of the North Pacific Subtropical Gyre—most primary production in the DCM (100–125 m depth) is accomplished by low-light-adapted *Prochlorococcus* ecotypes [8, 9] whose Fe requirements have not been characterized.

We quantified the Fe requirements of *Prochlorococcus* MIT1214, a member of the LL1 ecotype isolated from Station ALOHA, under a matrix of Fe and irradiance typical of the DCM (see Supplementary Materials and Methods), which follows the 0.5 mol photon m^−2^ day^−1^ isolume [10] and overlaps with peak abundance of LL1 *Prochlorococcus* (0.1–1 mol photon m^−2^ day^−1^ [9]). Under Fe-replete conditions (>150 pM Fe′), specific growth rates (*μ*) of *Prochlorococcus* MIT1214 were dependent on irradiance (Fig. 1a), indicating that LL1 *Prochlorococcus* at the DCM are in a light-limited regime. At 1.7 mol photon m^−2^ day^−1^, growth became Fe-limited at a bioavailable iron concentration (Fe′) of 73 pM, which intensified at 33 pM Fe′ (Fig. 1b). These concentrations also limited growth at 0.86 mol photon m^−2^ day^−1^ but not at 0.22 mol photon m^−2^ day^−1^. Fe limitation at 0.22 mol photon m^−2^ day^−1^ was only observed below 20 pM Fe′. In all treatments, the Fe:C composition of harvested cells primarily reflected Fe′ (Fig. 1c), but greater Fe:C was needed for faster growth rates at greater irradiance. At saturating light (1.7 mol photon m^−2^ day^−1^), cells bearing an Fe:C ratio of 36 ± 6 × 10^−6^ were still Fe-limited while cells growing at low light (0.22 mol photon m^−2^ day^−1^) reached peak growth rate with an Fe:C ratio of 20 ± 2 × 10^−6^ (Table S1).

**Fig. 1:**
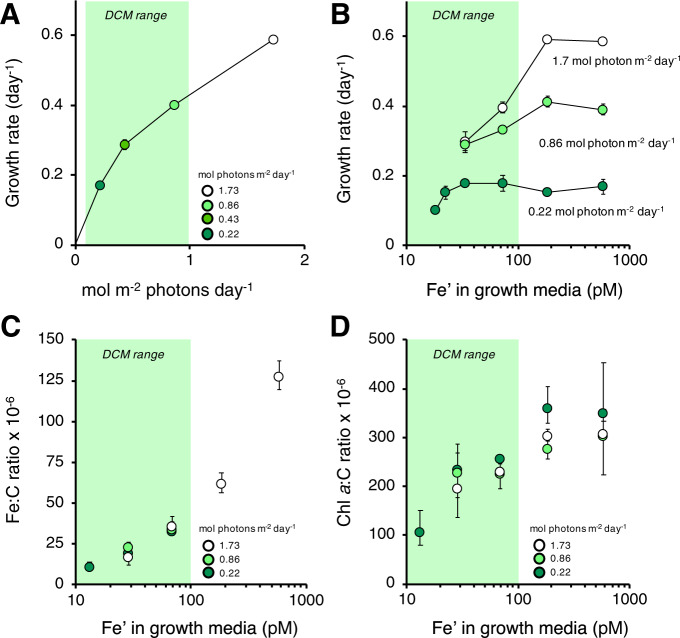
Growth of *Prochlorococcus* MIT1214  under low iron and low light. Specific growth rates as a function of **a** irradiance under Fe-replete conditions and **b** bioavailable Fe concentrations (Fe′). **c** The Fe:C ratio and **d** chlorophyll *a*:C ratio in harvested cells as a function of Fe′. Error bars reflect the range of triplicate measurements. Shading highlights typical irradiance [10] and dFe [6] at the DCM of Station ALOHA in the North Pacific Subtropical Gyre, where *Prochlorococcus* MIT1214 was originally isolated.

Smaller Fe requirements at lower irradiance is counter to models of Fe-light co-limitation, which is predicted from theory [3] and has been observed in model diatoms [4] and cyanobacteria [5]. In this state, either increased light or increased Fe can lead to higher growth rate: greater Fe supply will enable the construction of more photosynthetic units (PSU), allowing more light to be absorbed, increasing growth. Alternatively, an increase in irradiance will increase photon absorption with a fixed number of PSU. Fe-light co-limitation should manifest in the iron use efficiency (IUE) of growth, which describes the rate that cells accumulate biomass C per catalytic Fe atom. The IUE can be identified empirically as the slope between cellular Fe:C and *μ* under Fe-limiting conditions (Fig. 2). Fe-light co-limitation will cause the IUE to vary under different light regimes (Fig. 2a), with values proportional to the difference in photon flux [3]. In contrast, if Fe limitation and light limitation are independent physiological states (i.e. if low light does not increase Fe requirements), then a single IUE would apply for multiple light levels (Fig. 2b). Our observations of *Prochlorococcus* MIT1214 most closely reflect the latter scenario (Fig. 2c), which follow an IUE of 1.2 × 10^4^ mol C mol Fe^−1^ day^−1^. Thus, *Prochlorococcus* MIT1214 does not appear to be subject to Fe-light co-limitation under conditions relevant to the DCM.

**Fig. 2:**
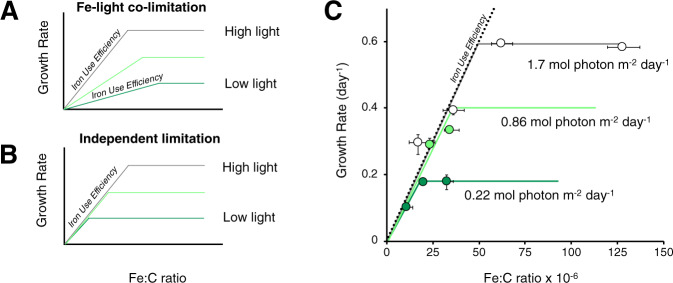
An empirical test of Fe-light co-limitation. Predicted relationships between Fe:C and growth rate under **a** a state of Fe-light co-limitation, or **b** independent states of light limitation and Fe limitation. The slope between growth rate and Fe:C corresponds to the iron use efficiency (IUE). **c** Observed relationships between growth rate and cellular Fe:C ratios for *Prochlorococcus* MIT1214 at 0.22 (dark green circles), 0.86 (light green), and 1.7 mol photon m^−2^ day^−1^ (white). Experimental data are consistent with predictions for independent states of Fe and light limitation, following an IUE of 1.2 × 10^4^ mol C mol Fe^−1^ day^−1^ (dotted black line).

The magnitude of IUE in *Prochlorococcus* MIT1214 is similar to both the theoretical IUE of a generic photoautotrophic cell (1.15 × 10^4^ mol C mol Fe^−1^ day^−1^ at 0.5 mol photon m^−2^ day^−1^ [3]) and the empirical IUE of temperate eukaryotic phytoplankton under light limitation (0.97 × 10^4^ mol C mol Fe^−1^ day^−1^ [4]). Convergence between these estimates suggests that theoretical IUE calculations apply to *Prochlorococcus* MIT1214 but, unlike classical descriptions of Fe-light co-limitation, the number of PSUs (and therefore the Fe requirement) is not downregulated in response to increases in light. This interpretation is supported by the <15% difference in chlorophyll *a*:C ratios of Fe-replete *Prochlorococcus* MIT1214 grown at 0.22 and 1.7 mol photon m^−2^ day^−1^ (Fig. 1d; Fe′ >150 pM; ANOVA with post-hoc Tukey test, *p* > 0.05), and by the much higher IUE achieved by the high-light adapted strain *Prochlorococcus* MIT9215 (1.5 × 10^5^ mol C mol Fe^−1^ day^−1^) when grown at greater irradiance [11].

The absence of Fe-light co-limitation has also been observed in Southern Ocean phytoplankton capable of producing large photosynthetic antennae (~2000 chlorophyll molecules per PSU) that enable very high IUE and Fe:C ratios below 3 × 10^−6^ [12]. Low-light-adapted *Prochlorococcus* construct considerably smaller antennae (272 chlorophyll per PSU; Table S2), perhaps due to photochemical inefficiencies at the very low irradiance and warmer temperatures found in the DCM of subtropical gyres [12]. Based on the size of these antennae (300 and 360 nm^2^ for PSII and PSI, respectively [13]), an estimated 4.5 μm^2^ of photosynthetic membrane in a low-light-adapted *Prochlorococcus* cell can be populated with a maximum of 6700 PSU containing 134,000 Fe atoms (assuming a 1:1 ratio of PSI:PSII and 20 Fe atoms per PSU, see Supplementary Information for full calculation). Thus, a cell with 4 fmol C (2.41 × 10^9^ atoms) and maximally upregulated PSU would be expected to have an Fe:C ratio of 56 × 10^−6^, which is similar to our measurements (although the latter also includes a small Fe requirement associated with respiration [3]). Under DCM conditions, it may not be possible for other ecotypes to achieve substantially lower Fe:C without first developing larger antennae, which has not been demonstrated. The genomes of *Prochlorococcus* MIT1214 and other LL1 isolates already contain seven copies of the *pcb* gene, encoding the chlorophyll binding proteins that compose the antennae to PSI and PSII, which is comparable to the eight copies in extremely low-light-adapted SS120 strain [13, 14]. Furthermore, the absence of two putative ferredoxins in LL1 genomes (Table S3), a feature shared by the ‘HNLC’ ecotypes [15], suggests that LL1 *Prochlorococcus* have undergone some level of adaptation to low Fe in the lower euphotic zone.

In the North Pacific subtropical gyre, the abundance of LL1 *Prochlorococcus* peaks at 105 ± 18 m depth [9], where primary production is 0.17 ± 0.09 μM C day^−1^ (mean at 100 m at Station ALOHA). To avoid Fe limitation, our results indicate *Prochlorococcus* must maintain an Fe:C ratio above 30 × 10^−6^, requiring uptake of at least 5.1 ± 2.7 pM Fe day^−1^ from a small dFe inventory (64 ± 20 pM for 90–120 m [6]). Although this estimate can be further refined by characterizing co-occurring *Prochlorococcus* ecotypes and other phytoplankton found in the DCM, it implies that Fe in the lower euphotic zone turns over on the order of 13 ± 8 days (or less), substantially shorter than the 6–12 month residence time of dFe inferred at Station ALOHA [16]. Therefore, severe iron limitation could develop at the DCM if dFe is not recycled over a dozen of times before being scavenged or exported to depth. The need for high-efficiency Fe recycling likens the DCM to the Equatorial Pacific and other Fe-limited regions [17, 18], and may motivate the synthesis of siderophores in the lower euphotic zone [19].

## Supplementary information

Supplemental information
