# Supplementary material for: Independent iron and light limitation in a low-light-adapted Prochlorococcus from the deep chlorophyll maximum
Source: ISME J. 2020 Sep 23;15(1):359–62. doi: 10.1038/s41396-020-00776-y (PMC7852507; doi:10.1038/s41396-020-00776-y)
Supplement: Supplementary file 1 — Supplemental information [file 41396_2020_776_MOESM1_ESM.pdf]

Supplementary Information

Independent iron and light limitation in a low-light adapted *Prochlorococcus* from the deep chlorophyll maximum

Nicholas J. Hawco,[1†*] Feixue Fu,[2] Nina Yang,[2] David A Hutchins,[2] and Seth G. John[1]

Running title: Iron and light limitation in *Prochlorococcus*

[1] Department of Earth Sciences, University of Southern California, Los Angeles, CA
[2] Department of Marine and Environmental Biology, University of Southern California, Los Angeles, CA

[†] Present address: Department of Oceanography, School of Ocean and Earth Sciences and Technology, University of Hawai‘i at Mānoa, Honolulu, HI

*Corresponding author: hawco@hawaii.edu

Table of Contents

1. Supplementary Materials and Methods
2. Supplementary References
3. Supplementary Tables S1–S3

1. Supplementary Materials and Methods

Culturing procedures

Prochlorococcus MIT1214 was originally isolated from Station ALOHA in the North Pacific subtropical gyre (22.75ºN, 158ºW) in 2012 during the HOE-PHOR expedition from a depth of 175 m. Genomic analyses indicate that it belongs to the LL1 clade (also known as eNATL (1)). Cultures of *Prochlorococcus* MIT1214ax were provided by the Chisholm lab at MIT and grown at 18ºC under a 12:12 light dark cycle. Light levels in experiments were decreased using neutral shading. Prior to conducting iron limitation experiments, *Prochlorococcus* MIT1214 was acclimated to light levels corresponding to an instantaneous irradiance of 5, 10, 20, and 40 µmol photon m^{-2} s^{-1} (daily flux: 0.22, 0.44, 0.86, and 1.7 mol photon m^{-2} day^{-1}) in Pro99 media (2) in 50 mL polycarbonate bottles for at least one month.

During Fe gradient experiments, batch cultures were grown in 500 mL polycarbonate bottles (Nalgene) in a modified ProTM media (2,3). All bottles were washed by soaking

overnight in a 5% citranox solution, rinsing with ultrapure (18.2 MΩ) water, soaking in 10% hydrochloric acid for 1 week, and finally rinsed several times (>5x) in ultrapure water. Media was prepared with a seawater base obtained from 0.2 μm filtered water collected in the North Pacific subtropical gyre. Seawater was poured into 2L polycarbonate bottles (Nalgene) and microwave sterilized. Ammonium and phosphate sources were decreased from published recipes to 50 and 3.1 μM respectively. Macronutrient stocks were cleaned of contaminants using Chelex-100 resin (Bio-Rad) and all nutrient stocks were filter sterilized. Ethylene-diaminetetraacetic acid (EDTA) was added to a final concentration of 11.7 μM. Concentrations of inorganic iron (Fe') in equilibrium with EDTA were calculated as described in Sunda et al. (4), at a ratio of $10^{-1.94}$ relative to total Fe in the media. Added Mn, Co, Ni, and Zn concentrations were 20, 10, 8, and 10 nM respectively. Background Fe concentrations were measured to be 1.5 nM in background seawater by inductively coupled mass spectrometry (ICP-MS) following extraction using a SeaFAST preconcentration system (Elemental Scientific). Additional Fe was added as a 10 mM HCl solution and media was allowed to equilibrate overnight before cultures were inoculated.

Preliminary growth experiments at a given Fe concentration were conducted in 50 mL polycarbonate bottles. 5–10 mL of exponential phase cells were then inoculated into 500 mL bottles and growth was monitored by *in vivo* chlorophyll fluorescence at regular (48 hr) intervals. Approximately 4 mL of culture was poured into glass test tubes underneath a HEPA filter and then measured with a 10-AU fluorometer (Turner Designs). Specific growth rates were determined by the rate of increase in the natural log of *in vivo* chlorophyll fluorescence. Cultures were harvested during mid/late exponential growth.

Particulate carbon and chlorophyll analyses

Duplicate 30–50 mL aliquots of culture were filtered onto 25 mm GF/F filters (Whatman). For chlorophyll analyses, frozen filters were extracted in 90% acetone overnight at -20ºC and extracted chlorophyll was quantified on a 10-AU fluorometer. Prior to use, filters for particulate carbon were combusted at 500 ºC for >3 hours. After filtration, filters were dried over 48 hours at 60 ºC and then measured with a 4010 Elemental Analyzer (Costech), calibrated with methionine and acetanilide standards, as described previously (5).

Particulate metal analyses

For particulate metal analyses, 100–200 mL aliquots were filtered onto 47 mm 0.2 μm polyethersulfone membranes under vacuum in a class 1000 clean room. Filters were digested overnight at 95 ºC in 30 mL perfluoroalkoxy vials (Savillex) by refluxing 5 mL of 50% distilled nitric acid (HNO_3) with 1 ppb Indium added. Filters were then removed and samples were dried down at 100ºC and subsequently digested in 200 μL of 1:1 HNO_3:HCl for 2 hours. Digested metals were dried down again and re-dissolved in 0.1 M HNO_3 for analysis on an Element2 ICPMS (Thermo Fisher). Fe concentrations were determined in medium resolution mode relative to a standard curve of 0.1–100 ppb, diluted from a certified reference (Inorganic Ventures). Intensity of the ^{115}In peak was used to correct for matrix effects, as well as sample loss during digestion (e.g. acid adsorbed onto the filter). Combined reagent and filter blanks (42 ± 21 pmol, n = 10) were quantified and subtracted from sample values (180 to 3,200 pmol). Fe concentrations were calculated after dividing by the volume filtered, which was determined gravimetrically.

We note that filters were not washed with chelating solutions prior to digestion. Initial experiments suggested a significant amount of cell lysis occurred during short (~5 min) rinses in an oxalate-EDTA solution. As a result, particulate Fe concentrations may contain some amount of Fe bound to the cell surface. Most of our experiments are well below the Fe hydroxide precipitation thresholds described by Sunda and Huntsman (500 pM Fe’ (6)), meaning that this effect is minor, especially when growth was Fe-limited.

Comparisons to HOT data

Primary production measurements for Hawaii Ocean Time-series cruises 1-298 were accessed via the HOT-DOGS website (hahana.soest.hawaii.edu/hot/hot-dogs/interface.html; ref. 7). Measurements made at 100 m (n = 273) were averaged. All dissolved iron data between 90 to 120 m from Fitzsimmons et al. (8) were averaged. It should be noted that there is a summertime bias in the dFe measurements at Station ALOHA, especially in this depth range. Because primary production at 100 m in summer will be on the higher end of the range quoted in text, the resulting turnover times may be on the lower end of the quoted range.

2. Supplementary References

1. Berube PM, Rasmussen A, Braakman R, Stepanauskas R, Chisholm SW. Emergence of trait variability through the lens of nitrogen assimilation in *Prochlorococcus*. Elife. 2019;8:e41043.
2. Moore LR, Coe A, Zinser ER, Saito M a., Sullivan MB, Lindell D, et al. Culturing the marine cyanobacterium *Prochlorococcus*. Limnol Oceanogr Methods. 2007;5(Table 1):353–62.
3. Hawco NJ, Saito MA. Competitive inhibition of cobalt uptake by zinc and manganese in a pacific *Prochlorococcus* strain : Insights into metal homeostasis in a streamlined oligotrophic cyanobacterium. Limnol Oceanogr. 2018;63:2229–49.
4. Sunda W, Price N, Morel F, Service NO, Road PI. Trace metal ion buffers and their use in culture studies. In: Algal Culturing Techniques. Academic Press; 2005. p. 35–63.
5. Fu F-X, Yu E, Garcia NS, Gale J, Luo Y, Webb EA, et al. Differing responses of marine N2 fixers to warming and consequences for future diazotroph community structure. Aquat Microb Ecol. 2014;72(1):33–46.
6. Sunda WG, Huntsman SA. Interrelated influence of iron, light and cell size on marine phytoplankton growth. Nature. 1997;390(6658):389–92.
7. Fujieki LA. Hawaiian ocean time-series data organization & graphical system (HOT-DOGS). 2007.
8. Fitzsimmons JN, Hayes CT, Al-Subiai SN, Zhang R, Morton PL, Weisend RE, et al. Daily to decadal variability of size-fractionated iron and iron-binding ligands at the Hawaii Ocean Time-series Station ALOHA. Geochim Cosmochim Acta. 2015;171(August 2015):303–24.
9. Ting CS, Hsieh C, Sundararaman S, Mannella C, Marko M. Cryo-electron tomography reveals the comparative three-dimensional architecture of *Prochlorococcus*, a globally important marine cyanobacterium. J Bacteriol. 2007;189(12):4485–93.
10. MacGregor-Chatwin C, Jackson PJ, Sener M, Chidgey JW, Hitchcock A, Qian P, et al. Membrane organization of photosystem I complexes in the most abundant phototroph on Earth. Nat plants. 2019;5(8):879–89.
11. Bibby TS, Nield J, Partensky F, Barber J. Antenna ring around photosystem I. Nature. 2001;413(6856):590.

12. Bibby TS, Mary I, Nield J, Partensky F, Barber J. Low-light-adapted *Prochlorococcus* species possess specific antennae for each photosystem. Nature. 2003;424(6952):1051–4.
13. Raven JA. The iron and molybdenum use efficiencies of plant growth with different energy, carbon and nitrogen sources. New Phytol. 1988;109(3):279–88.
14. Raven JA. Predictions of Mn and Fe use efficiencies of phototrophic growth as a function of light availability for growth and of C assimilation pathway. New Phytol. 1990;116(1):1–18.
15. Bertilsson S, Berglund O, Karl DM, Chisholm SW. Elemental composition of marine *Prochlorococcus* and *Synechococcus*: Implications for the ecological stoichiometry of the sea. Limnol Oceanogr. 2003;48(5):1721–31.
16. Moore LR, Rocap G, Chisholm SW. Physiology and molecular phylogeny of coexisting *Prochlorococcus* ecotypes. Nature. 1998;393(6684):464–7.

3. Supplementary Tables

Table S1. Experimental data for the *Prochlorococcus* MIT1214 Fe-light matrix

Instantaneous Irradiance, μmol photon m^{-2} s^{-1}	Daily Irradiance, mol photon m^{-2} day^{-1}	Added Fe, nM	Media Fe, nM	Media Fe', pM		μ, day^{-1}	Cell Carbon, μM	Cell Fe, nM	Fe:C ratio, x 10^{-6}	Chl *a*, μg L^{-1}	*In vivo* Chl Fluor., RFU	Chl *a*:C ratio, x 10^{-6}
5	0.216	0.15	1.65	13.2	A	0.10	109	0.96	8.8	7.82	0.23	80
5	0.216	0.15	1.65	13.2	B	0.10	92	0.85	9.3	7.12	0.20	87
5	0.216	0.15	1.65	13.2	C	0.11	63	0.89	14.1	8.4	0.24	150
5	0.216	0.5	2	17.2	A	0.13	*	1.23	*	12.4	0.28	*
5	0.216	0.5	2	17.2	B	0.15	*	1.19	*	10.1	0.24	*
5	0.216	0.5	2	17.2	C	0.17	*	1.24	*	10.9	0.24	*
5	0.216	1.5	3	28.7	A	0.18	109	1.93	17.6	21.5	0.52	221
5	0.216	1.5	3	28.7	B	0.18	92	1.92	20.8	23.5	0.58	286
5	0.216	1.5	3	28.7	C	0.18	91	1.85	20.4	15.6	0.42	193
5	0.216	5	6.5	68.9	A	0.20	111	3.44	30.9	15.6	0.61	158
5	0.216	5	6.5	68.9	B	0.16	100	3.06	30.6	25.3	0.49	284
5	0.216	5	6.5	68.9	C	0.18	79	2.85	35.9	21.9	0.44	309
5	0.216	15	16.5	183.7	A	0.15	92.2	na	na	33.3	0.69	405
5	0.216	15	16.5	183.7	B	0.16	87.7	na	na	25.7	0.55	329
5	0.216	15	16.5	183.7	C	0.15	126.1	na	na	38.7	0.85	345
5	0.216	50	51.5	585.6	A	0.16	100.4	na	na	31.9	0.53	357
5	0.216	50	51.5	585.6	B	0.17	81.5	na	na	25.9	0.67	357
5	0.216	50	51.5	585.6	C	0.17	112.4	na	na	33.3	0.70	333
10	0.432	15	16.5	183.7	A	0.29	154.4	na	na	46.2	1.08	336
10	0.432	15	16.5	183.7	B	0.30	152.3	na	na	54.3	1.19	400
10	0.432	15	16.5	183.7	C	0.27	126.8	na	na	43.2	0.93	382
10	0.432	50	51.5	585.6	A	0.31	240.8	na	na	70.2	1.83	327
10	0.432	50	51.5	585.6	B	0.27	142.8	na	na	48.9	1.13	384
10	0.432	50	51.5	585.6	C	0.29	164.4	na	na	63.3	1.41	432
20	0.864	1.5	3	28.7	A	0.31	86.5	2.1	24.4	13.7	0.32	177
20	0.864	1.5	3	28.7	B	0.28	93.2	2.1	22.1	22.3	0.56	268
20	0.864	1.5	3	28.7	C	0.28	94.9	**	**	19.9	0.56	235
20	0.864	5	6.5	68.9	A	0.33	94.4	3.0	31.9	16.5	0.51	196
20	0.864	5	6.5	68.9	B	0.34	107.9	4.2	39.1	24.1	0.78	251
20	0.864	5	6.5	68.9	C	0.33	137.9	4.3	31.1	28.6	0.92	232
20	0.864	15	16.5	183.7	A	0.42	88.5	na	na	20.2	0.40	256
20	0.864	15	16.5	183.7	B	0.39	66.4	na	na	16.5	0.33	279
20	0.864	15	16.5	183.7	C	0.42	71.2	na	na	18.7	0.38	294
20	0.864	50	51.5	585.6	A	0.40	97.5	na	na	19.9	0.41	229
20	0.864	50	51.5	585.6	B	0.40	93.1	na	na	18.6	0.39	224
20	0.864	50	51.5	585.6	C	0.37	43.1	na	na	17.4	0.35	453
40	1.728	1.5	3	28.7	A	0.26	107.8	1.3	12.0	13.1	0.30	136
40	1.728	1.5	3	28.7	B	0.31	131.2	1.6	12.4	26.9	0.63	230
40	1.728	1.5	3	28.7	C	0.32	88.8	2.3	26.2	17.5	0.37	221
40	1.728	5	6.5	68.9	A	0.38	113.8	4.0	35.5	22.1	0.50	217
40	1.728	5	6.5	68.9	B	0.40	134.4	4.1	30.6	28.3	0.61	236
40	1.728	5	6.5	68.9	C	0.40	108.8	4.6	41.9	22.6	0.49	233
40	1.728	15	16.5	183.7	A	0.60	191.1	13.1	68.3	54.0	1.29	317
40	1.728	15	16.5	183.7	B	0.59	201.4	12.2	60.5	54.0	1.24	301
40	1.728	15	16.5	183.7	C	0.59	203.2	11.5	56.5	52.8	1.24	292
40	1.728	50	51.5	585.6	A	0.58	221.0	27.8	125.9	58.8	1.27	299
40	1.728	50	51.5	585.6	B	0.59	215.0	25.7	119.4	59.1	1.25	309
40	1.728	50	51.5	585.6	C	0.58	200.1	27.4	137.1	55.8	1.16	313

* samples removed due to suspect POC measurements (near detection limit)

** sample removed due to Fe contamination (particulate Fe > media Fe)

Supplemental Table 2. Prediction of *Prochlorococcus* Fe requirements under low light (note that PSU describes a photosynthetic unit containing both photosystems I and II).

Parameter	Value	Units	Derivation (Reference)
Photosynthetic membrane area	4.5	μm^2 $cell^{-1}$	4 spherical membrane layers with R = 0.3 µm (Ting et al., 2007 (9))
PSI antennae size (per PSI trimer)	1089	nm^2	33 x 33 nm (Bibby et al., 2001 (11), 2003 (12))
PSII antennae size (per PSII dimer)	609	nm^2	21 x 29 nm (Bibby et al., 2003 (12))
PSI:PSII ratio	1		(Bibby et al., 2001 (11), Macgregor-Chatwin et al., 2019 (10))
Antennae area per PSU	668	nm^2	= 1089/3 + 609/2
PSU per cell	6700	$cell^{-1}$	= 4.5 x 10^6 / 668
Fe per PSU	20	atoms PSU^{-1}	Assuming electron transport by flavodoxin and plastocyanin (Raven 1988 (13), 1990 (14))
Photosynthetic Fe per cell	134,000	atoms $cell^{-1}$	= 6700 x 20
C per cell	2.41 x 10^9	atoms $cell^{-1}$	= 4 fmol C x 6.022 x 10^{23} (Bertilsson et al., 2003 (15))
Photosynthetic Fe:C	56 x 10^{-6}	mol mol^{-1}	= 1.34 x 10^5 / 2.41 x 10^9
Measured Fe:C (Fe limited)*	11–36 x 10^{-6}	mol mol^{-1}	Table S1
Chl per PSI trimer	570		100/PSI x 3 PSI + 15/pcb x 18 pcb (Bibby et al., 2001 (11))
Chl per PSII dimer	164		30/PSII x 2 PSII + 13/Pcb x 8 Pcb (Bibby et al., 2003 (12))
Chl per PSU	272		= 570/3 + 164/2
Chl per cell	1.82 x 10^6	$cell^{-1}$	= 272 x 6700
Chl:C ratio	760 x 10^{-6}	mol mol^{-1}	= 1.82 x 10^6 / 2.41 x 10^9
Measured Chl *a*:C (Fe replete)	330 x 10^{-6}	mol mol^{-1}	Mean of 15 and 50 nM Fe treatments, Table S1
Chl *a*:*b* ratio	0.6–1.2	mol mol^{-1}	(Moore et al. 1997 (16))
Estimated Chl:C (Fe replete)	530–730 x 10^{-6}	mol mol^{-1}	= 330 x 1.6, 330 x 2.2

Supplemental Table 3. Presence/absence comparisons of iron-related genes in *Prochlorococcus* MIT1214 and other strains. 'X' indicates presence of genes, as determined by protein BLAST searches, with cut-off e-values of 1E-10.

		HLII		HLI	LLI				LLII/III	LLIV	*Synechococcus*
Gene		AS9601	MIT9215	MED4	NATL2A	NATL1A	PAC1	MIT1214	SS120	MIT9313	WH5701
Periplasmic Fe binding (idiA)[1]	ABM70636.1	X	X	X	X	X	X	X	X	X	X
Possible Fe porin[1]	CAE20459.1	X	X		X	X	X	X	X	X	X
Hydroxylase / Fe uptake factor[1]	CAE20461.1	X	X		X	X	X	X	X	X	X
Ferric uptake regulatory family	CAE19096.1	X	X	X	X	X	X	X	X	X	X
Ferritin[1]	ABM70152.1	X	X	X	X	X	X	X	X	X	X
Flavodoxin[1]	CAE19630.1	X	X	X	X	X	X	X	X	X	
petE plastocyanin	ABM69923.1	X	X	X	X	X	X	X	X	X	X
petF ferredoxin[1]	ABM70836.1	X	X	X	X	X	X	X	X	X	X
Possible ferredoxin[2]	ABM69628.1	X	X	X					X	X	X
Possible ferredoxin[2]	ABM70602.1	X	X	X					X	X	X
Metal binding protein[2]	ABM70560.1	X		X	X						
Fructose bisphosphate aldolase[2]	ABM70128.1	X		X					X	X	X
Plastoquinol terminal oxidase[2]	ABM69653.1	X	X	X	X		X	X			
Nitrite reductase	CAE08992.1				X	X	X	X	X	X	X
Nitrate reductase	CAE08979.1						X				X
FeS oxidoreductase[1]	CAE21037.1	X	X	X	X	X	X	X	X	X	X
FeS oxidoreductase[2]	ABM70984.1	X	X	X	X	X	X	X	X	X	X
Bacterioferritin-comigratory	CAE18804.1	X	X	X	X	X	X	X	X	X	X
High light inducible protein	CAE21329.1	X	X	X	X	X	X	X	X	X	
Antennae protein PcbA[3]	AAP99827.1	X	X	X	X	X	X	X	X	X	
Antennae protein PcbB[3]	AAQ00214.1				X	X	X	X	X		
Antennae protein PcbC[3]	AAP99929.1	X	X		X	X	X	X	X		
Antennae protein PcbD[3]	AAQ00212.1				X	X	X	X	X		
Antennae protein PcbE[3]	AAQ00494.1				X	X	X	X	X		
Antennae protein PcbF[3]	AAQ00332.1				X	X	X	X	X		
Antennae protein PcbG[3]	AAP99936.1								X	X	
Antennae protein PcbH[3]	AAQ00219.1				X	X	X	X	X		
Photosystem II protein Y[2]	ABM70507.1	X	X	X							
Glycoprotein[2]	ABM69794.1	X	X	X							
Chorismate-binding enzyme[2]	ABM70550.1	X		X							
Fumarate reductase subunit D[2]	ABM70562.1	X		X	X						X
Heat labile enterotoxin alpha[2]	ABM70585.1	X	X	X							
Purine phosphoribosyltransferase[2]	ABM70350.1	X	X	X	X	X	X	X	X		
Arsenate reductase[2]	ABM69855.1	X	X	X	X	X	X	X	X	X	X
Hemagglutinin neuraminidase[1,2]	ABM70128.1	X	X	X	X	X	X	X	X		X
Transglutaminase-like[2]	ABM70428.1	X	X	X	X	X	X	X			X
Stress induced protein OsmC[2]	ABM70409.1	X	X	X	X	X	X	X	X	X	X

[1] Identified as significantly regulated or of interest in Fe limitation / rescue studies of MED4 or MIT9313 by Thompson et al. (2011).

[2] Absent from HNLC *Prochlorococcus* clades, as identified by Rusch et al. (2010)

[3] Gene names refer to SS120 strain as defined by Bibby et al. (2003). All *pcb* genes are highly similar (e-values <1E-100). Assignment of the seven LL1 *pcb* genes in MIT1214, NATL2A, NATL1A, and PAC1 is based on the annotations of the MIT1214 genome and may not be exact.
